# Evaluation of Mobile Intermittent Fasting Applications in Chinese App Stores: Quality Evaluations and Content Analysis

**DOI:** 10.2196/66339

**Published:** 2025-10-07

**Authors:** Laihao Fang, Cheng Huang, Bing Lin, Kuanlin Lei, Jiazhen Zhou, Xiaoni Zhong, Yanbing Liu, Jiaxiu Liu

**Affiliations:** 1 College of Artifical Intelligence Medicine, Chongqing Medical University, No.1 Medical College Road, Chongqing, 400016, China, 023 6848 0060; 2 Medical Data Science Academy, Chongqing Medical University, Chongqing, China; 3 School of Software, Shandong University, Jinan, China; 4 School of Public Health, Chongqing Medical University, Chongqing, China

**Keywords:** mHealth, mobile health, mobile applications, digital interventions, app evaluation, intermittent fasting, weight management, content analysis

## Abstract

**Background:**

Obesity and related disorders are rising globally, especially in China, where they are linked to chronic diseases like diabetes and cardiovascular issues. As intermittent fasting (IF) gains popularity for weight management, the use of IF apps has increased, yet their quality varies significantly. A systematic evaluation of these apps is essential to assess their effectiveness and reliability.

**Objective:**

This study aimed to conduct a comprehensive evaluation of IF apps available in the Chinese mobile app market. We concentrated on evaluating their features, quality, and overall user experience to help users avoid low-quality options and direct app developers to enhance their offers.

**Methods:**

A systematic search was performed across 5 major app stores in China, including the Apple App Store, Huawei AppGallery, Oppo Software Store, Vivo App Store, and Xiaomi Market. “Fasting”, “Intermittent Fasting”, “Time-Restricted Feeding”, “Time-Restricted Fasting”, “Time-Restricted Eating” and “Meal Skipping” were used as keywords to identify relevant apps, which were then screened based on inclusion and exclusion criteria. The evaluation was conducted using the user version of the Mobile Application Rating Scale (uMARS). The uMARS assessment examined 4 key subscales: engagement, functionality, aesthetics, and information. Each app was independently evaluated by 2 raters who underwent uniform training to ensure consistency in scoring.

**Results:**

A total of 35 apps were assessed for the study. These apps mostly contain features such as fasting timer (100.0%), recording weight (97.14%), fasting reminder (85.71%), and recording water intake (85.71%). All of the apps have an obvious privacy protection. Most of the apps (79%) have tools for quantifying users’ health status. The results showed that the overall average uMARS score across the apps was 4.35 (SD 0.51). The subscale scores were as follows: engagement 4.42 (SD 0.47), functionality 4.65 (SD 0.31), aesthetics 4.19 (SD 0.64), and information 4.15 (SD 0.58). The functionality subscale had the highest mean score, while the aesthetic subscale showed the greatest range of scores, from 2.17 to 5.00. The overall uMARS score was significantly positively correlated with the subscale scores (*r*=0.786‐0.953, *P*<.001). The user ratings in the app stores did not significantly correlate with the uMARS overall scores (*r*=−0.290, *P*=.091). Strong inter-rater reliability was confirmed by intraclass correlation coefficients (ICC=0.809‐0.909 across subscales).

**Conclusions:**

All the apps reveal high overall quality but gaps in professional engagement and social features. Limited clinical input may undermine the evidence-based accuracy and long-term applicability of some apps. Developers are encouraged to collaborate with health care professionals to enhance content reliability and incorporate social features to boost user engagement, while ensuring robust privacy protections and reasonable use of artificial intelligence.

## Introduction

Recent statistics indicate that overweight/obesity continues to rise globally, with more than 2 billion people, or 30% of the world’s population, already overweight [1], prompting increased public health concerns. Nowadays, China has the highest number of people who are overweight and obese [2]. Obesity is linked to numerous chronic health conditions, including cardiovascular disease, cancer, diabetes, osteoarthritis, and chronic kidney disease [3-6], making weight management a crucial public health priority. Dietary interventions are stepping into the spotlight as people seek effective ways to lose weight and improve overall health [7].

With the rise of digital health technologies, mobile health (mHealth) apps have become an increasingly popular tool for managing various health conditions and lifestyle modifications [8]. Among these, intermittent fasting apps have gained significant attention due to the growing interest in intermittent fasting as a method for weight management and metabolic health improvement. Intermittent fasting, characterized by alternating periods of eating and fasting [9], is purported to offer various health benefits, such as improvements in dyslipidemia, blood pressure, and longer-term effects on cardiometabolic health [10,11]. A meta-analysis examined a randomized controlled trial of 899 Chinese participants, found that intermittent fasting significantly reduced body weight and BMI and was effective in improving insulin resistance and lipid indices [12], which not only supports the potential of intermittent fasting as a weight management strategy in the Chinese population but also demonstrates its scientific validity as a metabolic health intervention. These findings confirm the metabolic modulatory effects of intermittent fasting at the level of biological mechanisms. In this context, global studies are deepening the understanding of intermittent fasting from multiple dimensions, including the exploration of its mechanisms, the validation of an application for the assessment of dietary compliance [13], retention rates, fasting patterns, and weight loss effects of an intermittent fasting app [14]. Moreover, a qualitative analysis of the descriptions of weight loss apps on the Spanish market showed that features like “quick results” and “personalized plans” dominate marketing, but often lack empirical support, suggesting gaps in the quality of the apps and the need for a systematic evaluation [15]. Current studies are limited to systematic evaluations of fitness and weight management apps or focus on single intermittent fasting applications, and there have been no systematic evaluations of multiple intermittent fasting applications [13,16-18]. The main features offered by intermittent fasting apps in the Chinese app market range from tracking fasting periods and providing reminders to providing educational content about intermittent fasting and nutrition.

Given the significant role that intermittent fasting apps can play in influencing users’ health behaviors and aiding the development and improvement of mHealth applications, it is crucial to evaluate their quality. Therefore, we used the user version of the Mobile Application Rating Scale (uMARS) because it is a widely recognized and validated tool specifically designed for assessing the quality of mobile health apps [19]. uMARS is an optimized and user-friendly version of the original Mobile Application Rating Scale (MARS). Its reliability and validity have been verified, demonstrating a high Cronbach α coefficient in different linguistic contexts [20-24] and various scenarios [25] such as weight management and health tracking applications.

This study seeks to deliver a comprehensive functional analysis and systematic evaluation of intermittent fasting applications available in the Chinese mobile application market. In 2025, designated as the “Year of Weight Management” for Chinese citizens, China is implementing a 3-year action plan for weight management as a part of “Healthy China 2030” national strategy [26]. Adopting a user-centered approach, this study evaluates these applications through subscales reflecting user needs, preferences, and viewpoints. This research on intermittent fasting apps in China App Stores has significant scientific value and practical guidance, helping users identify low-quality options, determine effectiveness for weight loss and health management, and enhance user overall experience. Additionally, evaluating app quality yields valuable data for academic research, thereby highlighting strengths, limitations, and potential for enhancement.

## Methods

### Ethical Considerations

According to Article 32 of the Ethical Review Measures for Human Life Science and Medical Research Involving Human Participants (2023), jointly issued by the National Health Commission, the Ministry of Education, the Ministry of Science and Technology, and the State Administration of Traditional Chinese Medicine, research in human life science and medical studies that utilizes legally obtained public data or data generated through observation without interfering with public behavior may be exempt from ethical review [27]. Upon verification, the research described in the manuscript conforms to the circumstances outlined in the aforementioned regulations for exemption from ethical review. This study involves no human participants, and all data used were collected from legally and publicly accessible sources without any interaction or intervention.

### Search Strategy

This study provides a functional analysis and systematic evaluation of intermittent fasting apps in the mobile phone markets of China on April 1, 2025. Huawei AppGallery, Oppo Software Store, VIVO App Store, and Xiaomi Market are the most widely used Android app markets in China [28]. Thus, we searched the Apple App Store (for iOS apps), Huawei AppGallery, Oppo Software Store, VIVO App Store, and Xiaomi Market (for Android apps).

We conducted keyword searches using “Fasting”, “Intermittent Fasting”, “Time-Restricted Feeding”, “Time-Restricted Fasting”, “Time-Restricted Eating” and “Meal Skipping” across each app store in Chinese terms, respectively, without logging into any user accounts. If an app was found in multiple app markets, the version with the highest user score was selected for evaluation.

### Eligibility

After screening out duplicate apps, we further screened applications using certain exclusion-inclusion criteria. All apps that met the exclusion-inclusion criteria were downloaded to the test device. Apps that did not function properly due to technical reasons after being downloaded were excluded from the evaluation.

We will include apps related to intermittent fasting, with fasting timer features and those that have been continuously updated since January 1, 2023, and will exclude apps if they (1) have a user score of less than 3 and (2) are not in Chinese.

### Data Extraction

For each eligible app, data were extracted using a predefined extraction form. The data collected included general information and features of apps. The platform, user ratings from the app markets, the number of downloads, privacy protection measures, and any evidence-based or professional background supporting the app were noted as general information. All of the retrieved data were then arranged for additional analysis into structured tables. Table 1 shows the data that will be extracted from the apps.

**Table 1.** Data that will be extracted from the apps.

Assessment measure	Definition
General information	
Platform	Store platform from which the app originates (eg, Apple App Store, Huawei AppGallery)
User ratings from the app markets	Average rating given by users in the app store (eg, star rating)
Number of downloads	The total number of times the app has been downloaded from the app store
Privacy protection	Applying measures to protect user data privacy
Evidence-based and professional background	Whether the content or methodology of the app is based on scientific evidence or developed by professionals in a relevant field
Features	
App features	Specific features provided by the app (eg,notifications, timer)
Quality	
Engagement	The extent to which the app captures the user’s interest and encourages continued use (the uMARS subscale)
Functionality	The effectiveness and efficiency with which the app performs its intended function (the uMARS subscale)
Aesthetic	Visual appeal and design quality of the app, such as color scheme and layout organization (the uMARS subscale)
Information	Accuracy and usefulness of the information provided by the app (the uMARS subscale)

### Quality Appraisal of Apps

The uMARS will be used to evaluate the quality of the included apps. It is a widely recognized tool specifically designed for assessing the quality of mobile health apps [19] has been shown to be useful for assessing various aspects of mHealth apps in areas such as chronic diseases [29-31], mental disorders [32], and nutrition [33-35], and has been translated into several languages [20-24]. The uMARS scale consists of 26 items divided into 5 different subscales: engagement, functionality, aesthetics, information, and subjective. The focus of this study was to provide an objective assessment of the quality of the apps and therefore did not include the evaluation of subjective scale. Each item is rated on a 5-point Likert scale. The overall score is the mean of the averages of the 4 objective subscales. The reliability of the two raters’ scores will also be assessed.

Before formal scoring, the two raters underwent a uniform training supervised by a senior researcher with more than 5 years of expertise in digital health evaluation and preassessment of the applications and discussed inconsistencies in the results to ensure a common understanding of the criteria for uMARS. During the evaluation process, each app was evaluated independently by both raters, with at least 15 minutes of usage before scoring.

### Statistical Analysis

Mean values and SDs were used to describe quantitative variables in the uMARS in the qualitative evaluation. Frequencies and percentages were used to describe classification variables such as the features of the apps. Pearson correlation was calculated to compare the uMARS score with each subscale score, the uMARS score with user ratings, and the user ratings with each subscale score. The intraclass correlation coefficient (ICC) was calculated to measure the consistency of ratings across the subscales (engagement, functionality, aesthetics, and information) and the overall score. The ICC is a commonly used statistical method for assessing the reliability between raters [36]. All statistical analyses were performed using IBM SPSS Statistics 26. All figures were performed using PyCharm 2024.1.

## Results

### App Selection

**Figure 1.**
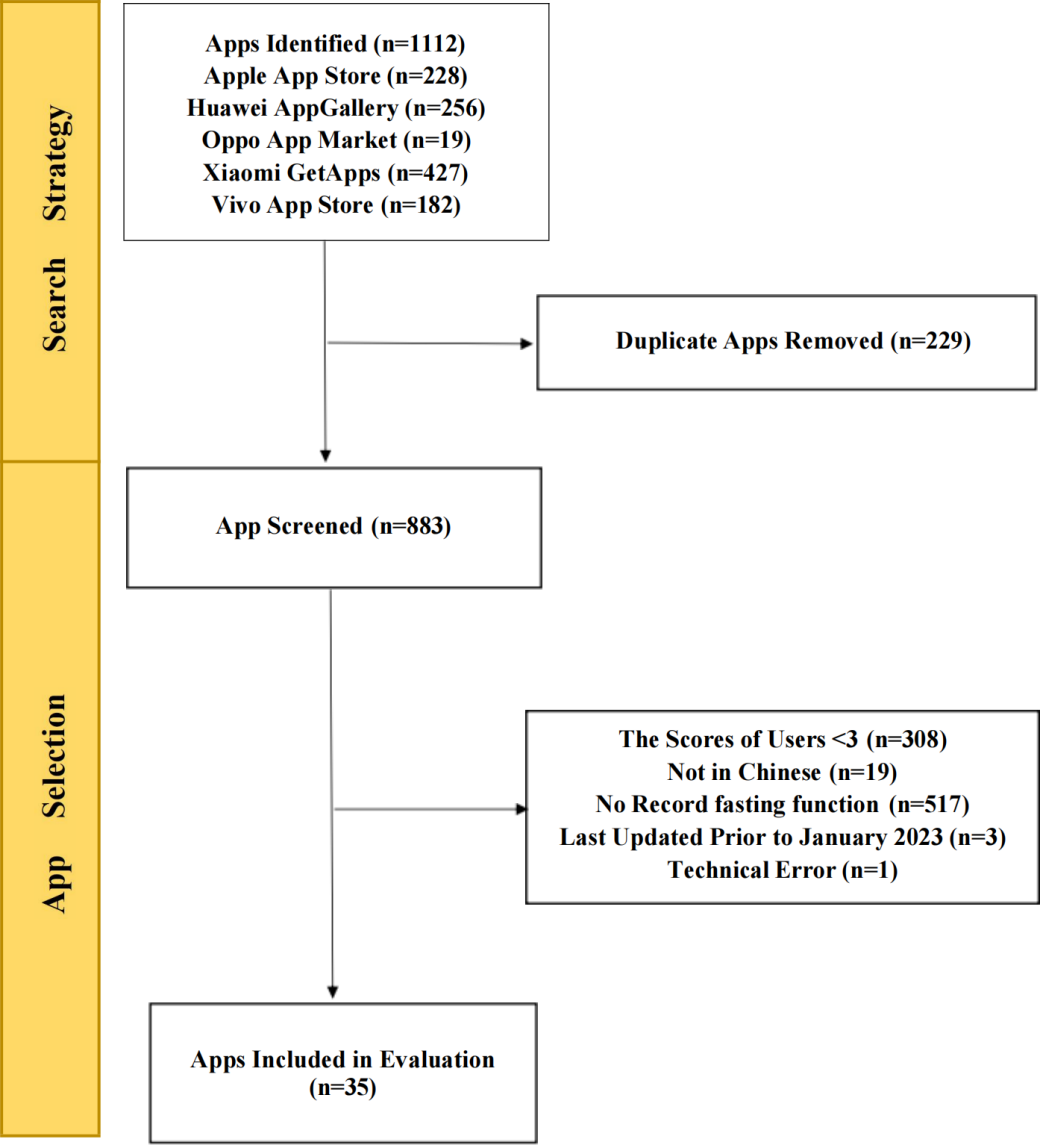
Flowchart for the systematic search and selection of apps.

A total of 1112 apps were identified from the Apple App Store (n=228), Huawei AppGallery (n=256), Oppo App Market (n=19), Xiaomi GetApp (n=427), and Vivo App Store (n=182). Combining the search results from the 5 app stores, we excluded 229 duplicate apps. A total of 848 apps were excluded on the basis of the exclusion-inclusion criteria. The remaining 35 apps were downloaded for evaluation. Figure 1 shows a flowchart of the intermittent fasting app selection process.

### General Information

Of the 35 apps included in the study, 12 (34%) were from the Apple App Store, 10 (29%) were from Xiaomi GetApp, and 10 (29%) were from the Vivo App Store. Only 2 (6%) apps were from the Huawei AppGallery, and another 1 (3%) app was from the Oppo App Market.

In terms of privacy protection, all 35 (100%) apps have obvious privacy protection measures in place.

Regarding the evidence-based and professional background, 11 (31%) apps have clinical physicians, nutritionists, and dietary experts involved in them. A total of 23 (65%) apps are supported by peer-reviewed academic research, ensuring the reliability of the content provided, and a total of 28 (80%) apps include tools for quantifying users’ health status. Table 2 shows the data that were extracted from the apps by analyzing apps store descriptions, in-app information, and developer statements.

**Table 2.** Data that were extracted from the apps.

Assessment measure	N (%)
Platform	
Apple App Store	12 (34)
Huawei AppGallery	2 (6)
Oppo App Market	1 (3)
Xiaomi GetApp	10 (29)
Vivo App Store	10 (29)
Privacy protection	
An obvious privacy protection	35 (100)
No privacy protection	0 (0)
Evidence-based and professional background	
Involvement of clinical physicians, nutritionists, and dietary experts	11 (31)
Peer-reviewed academic research supporting the content’s reliability	23 (65)
Tools for quantifying users’ health status	28 (80)

### Features of the Included Apps

**Figure 2.**
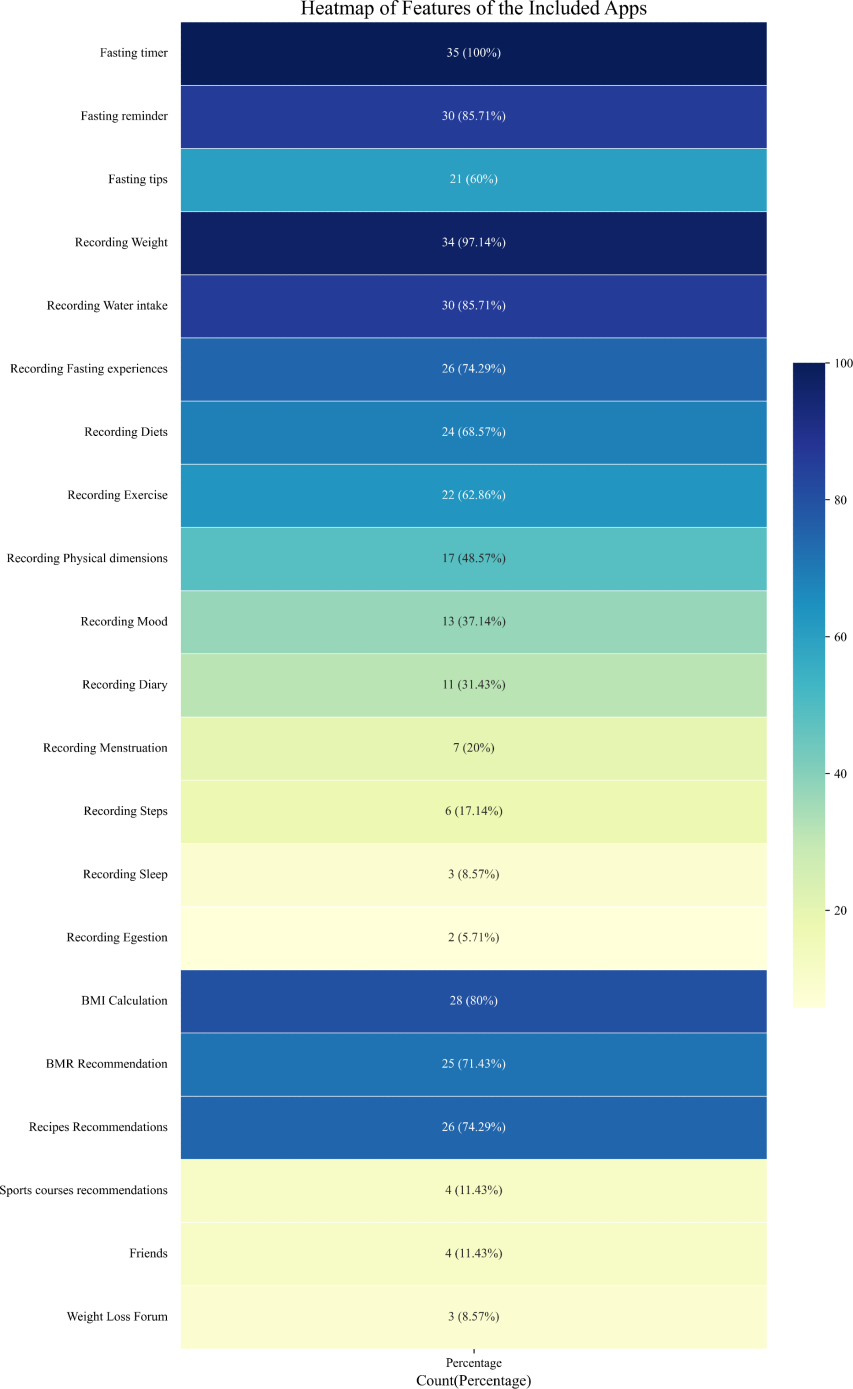
Heatmap showing the main functionalities of the intermittent fasting apps (n=35).

Of the 35 intermittent fasting apps downloaded, 100% (35/35) contain a fasting timer function, 85.71% (30/35) contain a fasting reminder function, and 60% (21/35) contain a provision of fasting tip functions. In addition to the abovementioned core functions, the features of these apps include 4 major categories: recording, calculation, recommendation, and socialization. Among the functions in the recording category are recording weight (34/35, 97.14%), water intake (30/35, 85.71%), fasting experiences (26/35, 74.29%), diet (24/35, 68.57%), exercise (22/35, 62.86%), physical dimensions (17/35, 48.57%), mood (13/35, 37.14%), diary (11/35, 31.43%), menstruation (7/35, 20%), steps (6/35, 17.14%), sleep (3/35, 8.57%), and egestion (2/35, 5.71%). For calculation functions, there are BMI calculation (28/35, 80%) and BMR calculation (25/35, 71.43%). The recommendation function has recipe recommendations (26/35, 74.26%) and sports courses recommendations (4/35, 11.43%), and the social function has a weight loss forum (3/35, 8.57%) and friend connections (4/35, 11.43%). Notably, several apps incorporate artificial intelligence (AI)-driven modules for automated diet and exercise and personalized meal planning. Figure 2 shows the main functionalities and their percentages. For more detailed information on the apps’ features, please refer to Multimedia Appendix 1.

### App Quality Score

Table 3 presents the final scores, mean values, and SDs for the following: (1) 4 subscales of the uMARS (engagement mean, functionality mean, aesthetic mean, and information mean), (2) app quality mean score (mean of 4 subscales), and (3) user ratings from the app markets.

**Table 3.** The user version of the Mobile Application Rating Scale mean scores for intermittent fasting apps^a^.

App	Engagement	Functionality	Aesthetic	Information	uMARS^b^	User ratings^c^
Boohee Health	4.90	4.88	4.67	4.63	4.77	4.70
Monster Intermittent Fasting^d^	4.70	4.88	4.33	4.63	4.63	4.70
Lanren Health	4.40	4.63	4.17	4.38	4.39	5.00
Lemon Fasting^d^	4.80	4.88	4.83	4.38	4.72	4.50
Grapefruit Intermittent Fasting^d^	5.00	5.00	5.00	4.88	4.97	4.20
Bigu Intermittent Fasting^d^	4.90	5.00	4.67	4.75	4.83	4.70
Fasting	4.80	5.00	4.50	4.50	4.70	3.80
Gugu Fasting^d^	5.00	4.75	4.83	4.75	4.83	4.90
FastingKeepFit	4.50	4.63	4.00	4.13	4.31	5.00
Health Fasting^d^	4.80	4.75	4.33	4.00	4.47	4.40
Fasting Bigu^d^	4.30	4.50	3.50	4.13	4.11	4.70
168 Intermittent Fasting App	4.80	5.00	4.50	4.50	4.70	4.80
Fasting now^d^	4.50	4.75	3.83	3.63	4.18	4.83
Weightloss Fasting	3.60	4.00	3.33	3.38	3.58	4.80
Tomato Health	4.90	4.88	5.00	5.00	4.94	4.60
Fasting Go^d^	4.90	4.75	4.50	4.50	4.66	4.62
Miaomiao Foods	4.80	4.38	4.83	4.75	4.69	4.81
Xifengyinlu Fasting^d^	3.90	4.75	4.00	3.38	4.01	4.93
Bohe Fasting^d^	4.30	4.88	3.83	3.88	4.22	5.00
Fasting Tracker	3.50	3.63	2.67	2.75	3.14	5.00
Nectarine Fasting^d^	4.60	5.00	4.83	4.50	4.73	4.55
Bianshenxiu^d^	4.70	4.75	4.00	4.50	4.49	5.00
Fastin: Intermittent Fasting	4.60	5.00	4.17	4.38	4.54	4.80
Fasting Tracker	3.20	4.13	2.17	2.38	2.97	4.50
Fasting Weightloss	3.00	3.75	2.17	2.88	2.95	5.00
Daily Fasting^d^	3.80	5.00	3.67	4.00	4.12	4.00
LemonFast	5.00	4.75	5.00	4.63	4.84	3.70
Diet Plan^d^	4.70	4.38	4.00	4.63	4.43	5.00
BodyOK	4.00	4.88	4.33	3.63	4.21	4.90
Fasting Timer^d^	3.80	4.63	4.50	4.25	4.29	4.80
Fasta Fasting Tracker	4.10	4.50	4.50	4.00	4.28	4.80
FastEasy	4.80	4.38	5.00	4.50	4.67	4.30
Shiguang Fasting^d^	4.30	4.63	4.33	4.00	4.31	4.10
Window - Fasting Tracker	4.60	4.63	4.17	4.00	4.35	4.00
Tomato Fasting^d^	4.20	4.38	4.33	4.25	4.29	4.70
Means	4.42	4.65	4.19	4.15	4.35	4.63
SD	0.47	0.31	0.64	0.58	0.46	0.36

a All items were rated on a 5-point scale from 1=inadequate to 5=excellent.

b The user version of the Mobile Application Rating Scale.

c The user ratings from the app markets.

d The app does not have an official English name, which is a literal translation in Chinese.

The overall average uMARS score of all apps was 4.35 (SD 0.46), with scores ranging from 2.95 (Fasting Weightloss) to 4.97 (Grapefruit Intermittent Fasting). The reliability of the uMARS is calculated as Cronbach α=.959. The uMARS score of 15 of 35 (42.9%) apps was ≥4.50. There are 16 of 35 (45.7%) apps with a uMARS score between 4.50 and 4.00. Furthermore, 2 of 35 apps (5.7%) had uMARS scores ranging from 3.00 to 3.99. And uMARS scores for 2 of 35 apps (5.7%) fell within the range of 2.90-2.99. There were no apps with a score of <2.90.

**Figure 3.**
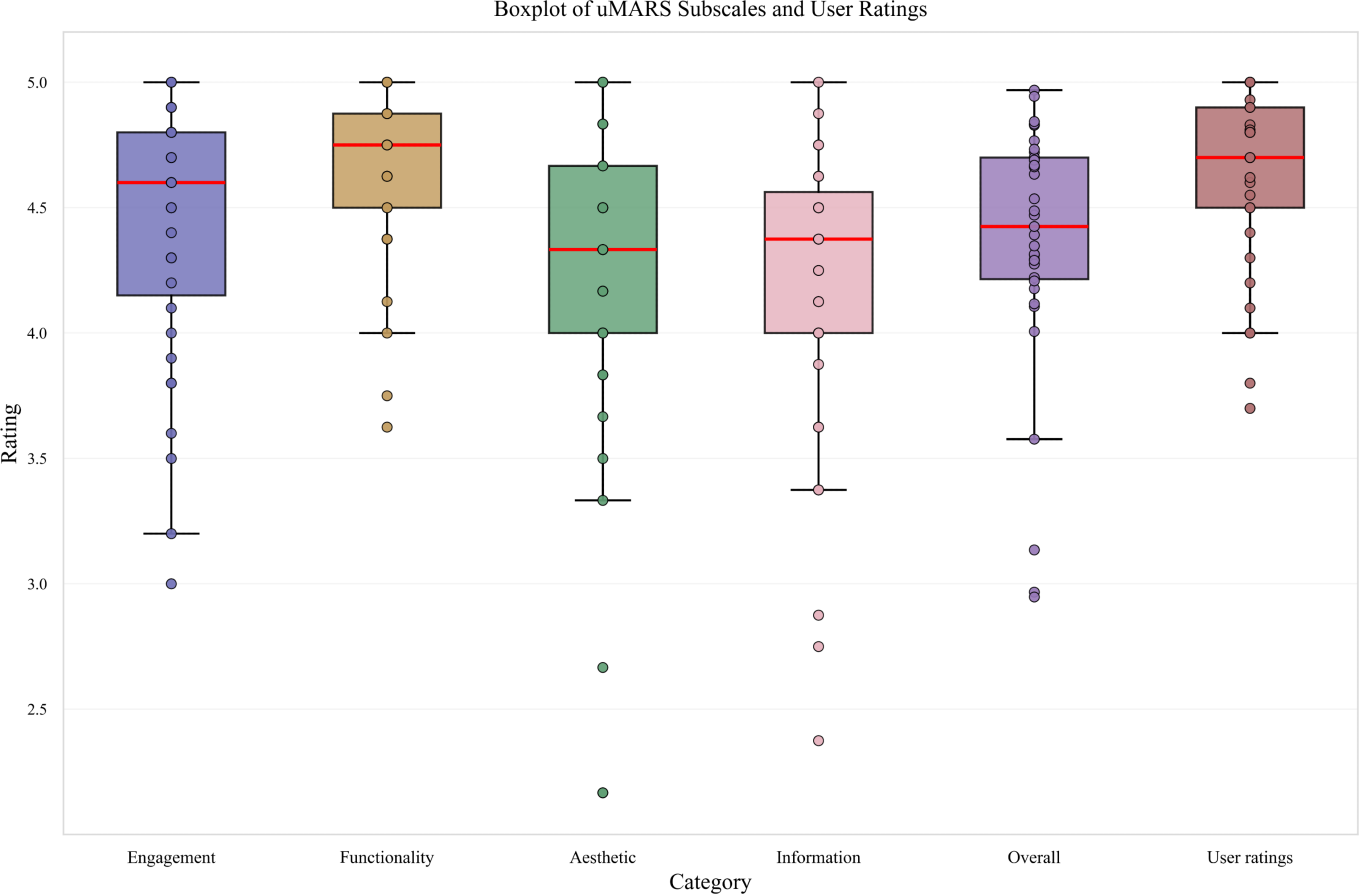
Graphical representation of the distribution of the uMARS overall, subscale score, and the user ratings. The median, the interquartile distance, and the range were given (n=35). uMARS: the user version of the Mobile Application Rating Scale.

The mean scores of each subscale were as follows: engagement quality score=4.42 (SD 0.47), functionality quality score=4.65 (SD 0.31), aesthetic quality score=4.19 (SD 0.64), and information quality score=4.15 (SD 0.58). The aesthetic quality scores showed the greatest span, ranging from a minimum of 2.17 to a maximum of 5.00. The distribution of scores for overall quality and the 4 subscale dimensions is shown in Figure 3.

### Correlation and Reliability

**Figure 4.**
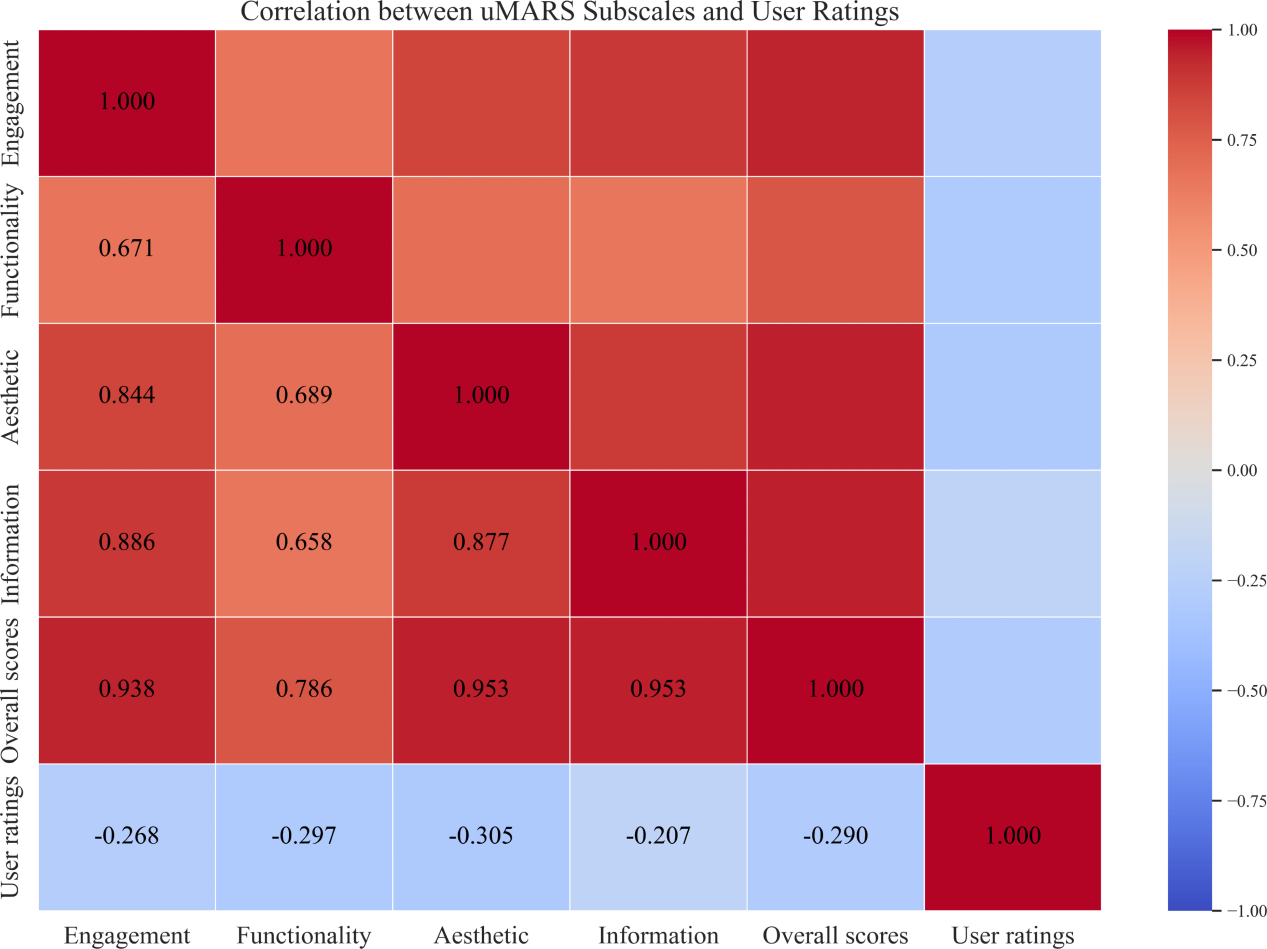
Heatmap showing the correlation between the user version of the Mobile App Rating Scale subscale and the overall score and user ratings.

Table 4 and Figure 4 show the correlation between the uMARS subscales and the overall score and user ratings. The overall uMARS score was significantly positively correlated with the subscale scores (*r*=0.786‐0.953, *P*<.001). However, user ratings of the app market did not correlate with either the overall uMARS score (*r*=−0.290, *P*=.091) or the subscale scores (*r*=-0.305‐-0.207, *P*=.075-.233).

**Table 4.** Correlation between the user version of Mobile App Rating Scale subscale and the overall score and user ratings.

Characteristic	Engagement	Functionality	Aesthetic	Information	Overall scores
Engagement
*P* value	__^a^				
Functionality
*P* value	0.671 (<.001)	__			
Aesthetic
*P* value	0.844 (<.001)	0.689 (<.001)	__		
Information
*P* value	0.886 (<.001)	0.658 (<.001)	0.877 (<.001)	__	
Overall scores
*P* value	0.938 (<.001)	0.786 (<.001)	0.953 (<.001)	0.952 (<.001)	__
User ratings
*P* value	−0.268 (.119)	−0.297 (.084)	−0.305 (.075)	−0.207(.233)	−0.290 (.091)

a Not applicable

The overall uMARS score showed high reviewer reliability (ICC 0.909, 95% CI 0.788‐0.957). Also, all subscales showed good consistency: engagement ICC 0.855 (95% CI 0.715‐0.927), functionality ICC 0.880 (95% CI 0.758‐0.940), aesthetics ICC 0.877 (95% CI 0.758‐0.938), and information ICC 0.809 (95% CI 0.537‐0.912). Table 5 shows the ICC between the Mobile App Rating Scale subscale and the overall score, as measured by the 2 raters.

**Table 5.** Intraclass Correlation Coefficient between the Mobile App Rating Scale subscale and the overall score, as measured by the 2 raters.

	Intraclass correlation^a^	95% CI		F test with true value 0			
		Lower bound	Upper bound	Value	df1	df2	*P* value
Engagement	0.855	0.715	0.927	6.932	34	34	<.001
Functionality	0.880	0.758	0.940	8.969	34	34	<.001
Aesthetic	0.877	0.758	0.938	8.370	34	34	<.001
Information	0.809	0.537	0.912	6.533	34	34	<.001
Overall	0.909	0.788	0.957	13.098	34	34	<.001

a Type A intraclass correlation coefficients using an absolute agreement definition.

## Discussion

### Principal Findings

This study aimed to provide a comprehensive functional analysis and systematic evaluation of intermittent fasting apps available in the Chinese market. We analyzed a total of 35 intermittent fasting apps, examining the various features, privacy protection, evidence-based support, and quality of each app.

Our results showed that of all 35 apps, almost all of them have a fasting timer (35/35, 100%) and the ability to record weight (34/35, 97.14%), and most offered water intake recording (30/35, 85.71%), BMI calculation (28/35, 80%), recipe recommendations (26/35, 74.29%), fasting experience recording (26/35, 74.29%), BMR calculation (25/35, 71.43%), and more. The presence of these features suggests that most apps are able to enable users to achieve their fasting plans by providing guidance and features required for fasting during the user’s weight loss phase, such as self-tracking, controlling dietary intake, and improving adherence to self-monitoring eating behaviors, which are important to support the user’s health goals [9,37]. Existing research suggests that social media and teammates’ influence facilitate the weight management process to a certain extent [38-41]. But the discrepancy is that very few apps (4/35, 11.43%) are able to provide social features. Notably, studies have shown that social features have a significant motivational effect on users with normal or low BMI but may trigger negative emotions in users with a higher BMI [39]. Thus, the low prevalence of social features may reflect developers’ considerations regarding user privacy protection and individual differences. Meanwhile, we noticed that some apps introduced AI tools to record diet, exercise, and customize recipes, etc. Some studies have shown that the introduction of AI modules indeed enhances user engagement and opens up new opportunities for apps, but the risks of AI for user privacy and algorithmic errors remain unpredictable to us [42,43].

Privacy protection measures were evident in all apps, reflecting the developers’ sensitivity to health data. A study has shown that users are more likely to use apps that clearly state privacy policies and data handling practices [44]. This is especially important for intermittent fasting apps as they collect sensitive data such as weight, eating habits, and fasting times. In addition to these, users are more afraid of receiving more personalized advertisements than the misuse of personal health data [45]. This suggests that the boundaries regarding the extraction of personal information also need to be rethought by developers.

Regarding evidence-based support, most of the apps provide tools to quantify the state of physical health and are backed by peer-reviewed academic research, ensuring the reliability of the content provided [46]. But there is evidence in the literature that app developers lack a comprehensive understanding of study design in translating scientific evidence into practical functionality, ignoring possible side effects or limitations. It is more appropriate for apps to focus on the longevity and completeness of the scientific intervention with applicability to different populations; otherwise, it may lead to misleading user behavior [47-50]. However, only a few apps claimed that their content had been developed with the involvement of clinicians, nutritionists, and dietary experts. This highlights a common flaw in the development process of intermittent fasting apps, which is the lack of the direct involvement of professionals with a systematic validation process, in line with previous literature findings, and such a shortcoming may affect the clinical accuracy and practical application of the information provided [51]. Weight loss applications should emphasize the importance of collaborative development with clinicians, nutritionists, and dietary experts and the need for their involvement early in the development process to ensure that the content is consistent with clinical practice guidelines and evidence-based medical standards [52-55].

The overall average uMARS score for all apps was 4.35 (SD 0.46), indicating high quality, which is consistent with international research performance [56]. The 3 apps with the highest uMARS quality score all received perfect scores for aesthetics, and all of them were designed with some kind of fruit as a specific theme, which could appeal to the user. Unlike traditional pages, several apps designed their interfaces with a cute scene along with cute characters to attract users. In the 3 apps with the lowest scores, both the aesthetic and information scores showed lower scores, which may also suggest a lack of evidence-based content for the apps [57,58]. The above indicates that it is crucial to balance aesthetics and information quality when designing apps [59]. In particular, the functionality subscale score of 4.65 (SD 0.31) is in line with the results of some previous literature, which found that the effectiveness of mobile apps in weight management is closely related to the quality of their design and that mobile app interventions can significantly enhance users’ physical activity levels and weight loss [60,61].

The various subscales in this study showed consistency with the total scale, and there is observational research that further suggests that frequency of app use and engagement are important factors in weight management success, which coincides with the positive correlation between engagement and overall score found in this study. These consistencies suggest that high-quality functional design and user engagement are key to making intermittent fasting apps work [62].

There was no significant correlation between user ratings in the app market and uMARS scores, which is consistent with existing research that suggests that user ratings may not always reflect the actual quality of the apps assessed by the standardized tool [25,63]. They pointed out that scores from app markets are usually based on the subjective experience of users and may be influenced by user preferences, advertisements, and other factors. A meta-analytic study showed that influencer marketing significantly affects consumer attitudes and behavioral engagement [64], and research has also shown that apps improve ratings by improving the user experience through fun or customization [65]. Also, developers may use bots or puppet accounts to post fake reviews [66]. These behaviors may result in high user ratings but not actual quality, and many user reviews are of low quality and hardly reflect the actual quality of the app [67]. Whereas the uMARS is an objective, professional assessment tool, this may lead to inconsistencies between user ratings and actual app quality. Similarly, a randomized controlled trial showed that app-based multimodal interventions were effective for weight management, but user ratings were not associated with the actual effects [68]. Meanwhile, our study found that the selected high user-rated health apps exhibit 2 key characteristics: systematic assessment scores on the uMARS are generally lower than consumer-side user ratings, and there is a notable lack of involvement of clinical medical experts in the product development process. This phenomenon echoes the findings of the Spanish language market study, which similarly found that weight loss apps, while achieving high user satisfaction, had a disconnect between their functional design and their scientific evidence base. Notably, both sets of studies revealed a common problem of existing apps in the market failing to effectively integrate specialized medical resources [69].

### Limitations and Perspectives

There are several limitations to consider. First, the keywords used in the app store searches may not have captured all relevant intermittent fasting apps as some may use different terms or be categorized differently, resulting in their exclusion from this study. Second, the app market is constantly evolving, with new apps being introduced and existing apps being updated or removed. The cross-sectional nature of this study means it provides a snapshot in time that may soon become outdated as the app landscape changes. Third, this study focused on the functionality and quality of the apps but did not measure long-term user engagement, adherence, or compliance with the apps, which are key factors in the success of a health intervention. Additionally, this study did not assess the actual effectiveness of the apps in promoting weight loss or improving metabolic health. The existence of a privacy policy does not necessarily guarantee that data protection measures are effectively enforced. Furthermore, the study evaluated only the free version of some of the apps due to lack of funding, and paying to provide additional functionality may have yielded different results. Last but not least, the subjective scale component of the uMARS was not included in this study when assessing app quality. Since subjective scales usually reflect users’ personal experiences and feelings, which may vary greatly depending on individual backgrounds, cultural differences, or other external factors. Meanwhile, the focus of the study is to provide an objective assessment of the functionality and quality of the applications, and the subjective feelings component may not directly affect the core objective of the study. Therefore, to ensure the objectivity of the data and the consistency of the assessment results, this study focuses on the objective dimensions of engagement, functionality, information, and aesthetic design of the apps.

Subsequent research will explore the long-term impact of using intermittent fasting apps on health outcomes, in addition to investigating user experience and engagement to understand the needs and preferences of different user groups to provide more targeted and effective information for development. Meanwhile, this research team is further expanding the evaluation team in upcoming studies to increase the data value of the study.

### Conclusions

This study identified 35 intermittent fasting apps in Chinese App Stores, analyzed the distribution of their main features, and assessed their content and quality. Most of the apps fulfill the basic requirements of intermittent fasting and are of high quality overall, but there are still gaps in terms of professional engagement and social features as well. Based on these findings, we recommend that developers consider engaging more health care professionals early in the app development process to ensure the content is more evidence-based and accurate. More evidence-based content will help enhance the informational quality of the apps, addressing the gaps identified in some lower-scoring apps. Furthermore, adding social features, such as community forums or friend connections, could increase user engagement and provide additional support, which has been shown to aid in weight management. By focusing on these areas, developers can create more comprehensive and user-friendly intermittent fasting apps that promote better adherence and outcomes for users. However, enhanced user social engagement should be accompanied by good user privacy protection to avoid counterproductive results. AI-driven tools have the potential to enhance user personalization, but they need to be used in reasonable moderation.

## Supplementary material

10.2196/66339 Multimedia Appendix 1 Main functionalities of the fasting apps (n=35).

## Abbreviations

AI: artificial intelligence
BMR: basal metabolic rate
ICC: intraclass correlation coefficient
the uMARS: the user version of the Mobile Application Rating Scale
