# Supplementary material for: Evaluation of Mobile Intermittent Fasting Applications in Chinese App Stores: Quality Evaluations and Content Analysis
Source: JMIR Mhealth Uhealth. 2025 Oct 7;13:e66339. doi: 10.2196/66339 (PMC12503444; doi:10.2196/66339)
Supplement: Multimedia Appendix 1 [file mhealth-v13-e66339-s001.docx]

| Table. Main functionalities of the fasting apps (n=35). | |  |
| --- | --- | --- |
| Functionalities | | N (%) |
| Core Functionality | Fasting timer | 35 (100.0) |
|  | Fasting reminder | 30 (85.71) |
|  | Fasting tips | 21 (60.0) |
| Recording | Weight | 34 (97.14) |
|  | Water intake | 30 (85.71) |
|  | Fasting experiences | 26 (74.29) |
|  | Diets | 24 (68.57) |
|  | Exercise | 22 (62.86) |
|  | Physical dimensions | 17 (48.57) |
|  | Mood | 13 (37.14) |
|  | Dairy | 11 (31.43) |
|  | Menstruation | 7 (20.0) |
|  | Steps | 6 (17.14) |
|  | Sleep | 3 (8.57) |
|  | Egestion | 2 (5.71) |
| Calculation | BMI^a^ | 28 (80.0) |
|  | BMR^b^ | 25 (71.43) |
| Recommendations | Recipes | 26 (74.29) |
|  | Sports courses | 4 (11.43) |
| Socialization | Friends | 4 (11.43) |
|  | Weight Loss Forum | 3 (8.57) |

^a^Body Mass Index

^b^Basal Metabolic Rate
